# Examining the Effects of Diet Composition, Soluble Fiber, and Species on Total Fecal Excretion of Bile Acids: A Meta-Analysis

**DOI:** 10.3389/fvets.2021.748803

**Published:** 2021-10-07

**Authors:** Julia Guazzelli Pezzali, Anna K. Shoveller, Jennifer Ellis

**Affiliations:** Department of Animal Biosciences, Centre for Nutrition Modelling, University of Guelph, Guelph, ON, Canada

**Keywords:** fat, protein, fiber, bile acid, fecal losses

## Abstract

Bile acids (BA) are produced in the liver and conjugated with glycine or taurine before being released into the small intestine to aid with lipid digestion. However, excessive BA losses through feces can occur due to several dietary factors that in turn require greater production of BA by the liver due to a reduction in BA recycling. Consequently, net utilization of taurine and/or glycine is increased. To quantify this impact, we conducted a meta-analysis to investigate the effect of soluble fiber, diet composition, and species on fecal excretion of BA. After a systematic review of the literature, twelve studies met all inclusion criteria. Dietary carbohydrate, protein, fat, cellulose, cholesterol, soluble fiber and animal species were tested as independent variables. Mixed models were developed treating study as a random effect, and fixed effect variables were retained at *P* < 0.05 significance and where collinearity was absent between multiple X variables. A total of ten studies comprised of four species [(rat = 5), hamster (*n* = 1), guinea pig (*n* = 3) and dog (*n* = 1)], and 30 observations were evaluated in the final models after outlier removal. Model evaluation was based on the corrected Akaike Information Criteria, the concordance correlation coefficient and the root mean square prediction error. Three base models were developed, examining carbohydrate, protein and fat impacts separately. The best fitting models included the fixed effect of species and the interaction between soluble fiber (yes/no) and dietary carbohydrate, protein or fat (%, as-fed). Increased concentrations of dietary protein and fat resulted in greater fecal excretion of BA (*P* < 0.05). Conversely, increasing levels of dietary carbohydrate led to lower excretions of BA (*P* < 0.05). Increased dietary soluble fiber containing ingredients resulted in greater excretion of BA in all models (*P* < 0.05). Rats had greater excretion of BA compared to hamsters and guinea pigs (*P* < 0.05) in all models, and also compared to dogs (*P* < 0.05) in the carbohydrate model. The findings from this meta-analysis indicate that not only soluble fiber, but also increasing levels of dietary fat and protein may result in greater fecal excretion of BA, potentially altering taurine and/or glycine metabolism and affecting the need for diet delivery of these AA.

## Introduction

Taurine deficiency has been previously reported as a causative factor for the development of dilated cardiomyopathy (DCM) in dogs (1) and cats (2, 3). Further to this, in 2018 the FDA also released a warning letter regarding a possible link between the development of DCM and dogs eating grain-free diets (4), where much attention was also placed on taurine. Taurine is a non-essential amino-sulfonic acid that is endogenously synthesized by the dog if sufficient amounts of its precursors, methionine and cysteine, are provided in the diet (5). Taurine also plays a major role in bile acid (BA) metabolism as dogs primarily conjugate BAs with taurine (6–8). As a result, losses of taurine can occur through the gastrointestinal tract and are directly related to BA losses.

Synthesis of BA occur in hepatocytes from cholesterol. In most species, they are stored in the gallbladder and released into the small intestine upon chyme arrival, to aid with digestion and absorption of lipids and fat-soluble vitamins (9). The secretion of BA is facilitated by cholecystokinin (CCK) that stimulates gallbladder contraction, and thus bile release (10). Prior to their secretion into the duodenum, BA must be conjugated with either glycine or taurine to increase their solubility and improve function as surfactants. The ratio of taurine to glycine-conjugates varies across species (11) and highlights one of the many species-specific differences regarding the metabolism of BA. In the terminal ileum, BA are resorbed by enterocytes and released in the portal vein to be transported back to the liver. This process is called enterohepatic circulation and recycles 95–98% of the total BA released in the small intestine (12). The BA that escapes reabsorption are excreted via feces. Interestingly, dietary fibers have been implicated in reduced BA reabsorption through binding with BAs, and have been shown to increase BA loss in the feces (13). Disruption of this recycling process not only impacts BA metabolism (e.g., resulting in increased BA production), but also glycine and taurine metabolism. It has been hypothesized that the high concentration of soluble fibers in grain-free diets, resulting from high inclusion of legumes, could lead to greater fecal losses of taurine (14) and may be the link to DCM. Measurements of fecal concentrations of taurine usually lead to an underestimation of its true content as gut bacteria in the large intestine can degrade taurine (15). Thus, fecal concentrations of BA are commonly used as biomarkers for fecal losses of taurine in dogs.

While the effect of soluble fibers on fecal concentrations of BA have been studied in rats (13, 16, 17), there is a dearth of data in dogs. Furthermore, even though both dogs and rats conjugate BA primarily with taurine, they have species-specific differences related to BA metabolism that may limit the translation of results among species. For example, rats do not possess a gallbladder and have a different composition of plasma BA compared to dogs (18). Ko et al. (19) were the first to report that beet pulp, a moderately fermented fiber, resulted in greater excretion of fecal BA in Beagle dogs. However, while similar results were observed by Donadelli et al. (20) when Labradors were fed a grain-free diet, Pezzali et al. (14) did not observe differences in total fecal excretion of BA in Beagles fed a grain-free compared to a grain-based diet. The inconsistency in results between studies raised two major questions: (1) are there any other dietary factors acting in conjunction with soluble fibers that could lead to greater excretion of fecal BA? and (2) can we translate the findings observed in rodent trials to dogs even with their differences in BA metabolism? Thus, the aim of this study was to use a meta-analysis approach to investigate the effect of soluble fiber inclusion, nutrient composition of the diet and species on total fecal excretion of BA while exploring other factors that could potentially contribute to this response. We hypothesized that: (1) increasing concentration of dietary fat, protein and soluble fiber would lead to greater fecal excretion of BA; and that (2) rats would have higher excretion fecal BA compared to the dog.

## Materials and Methods

### Literature Search and Database

**Figure 1:**
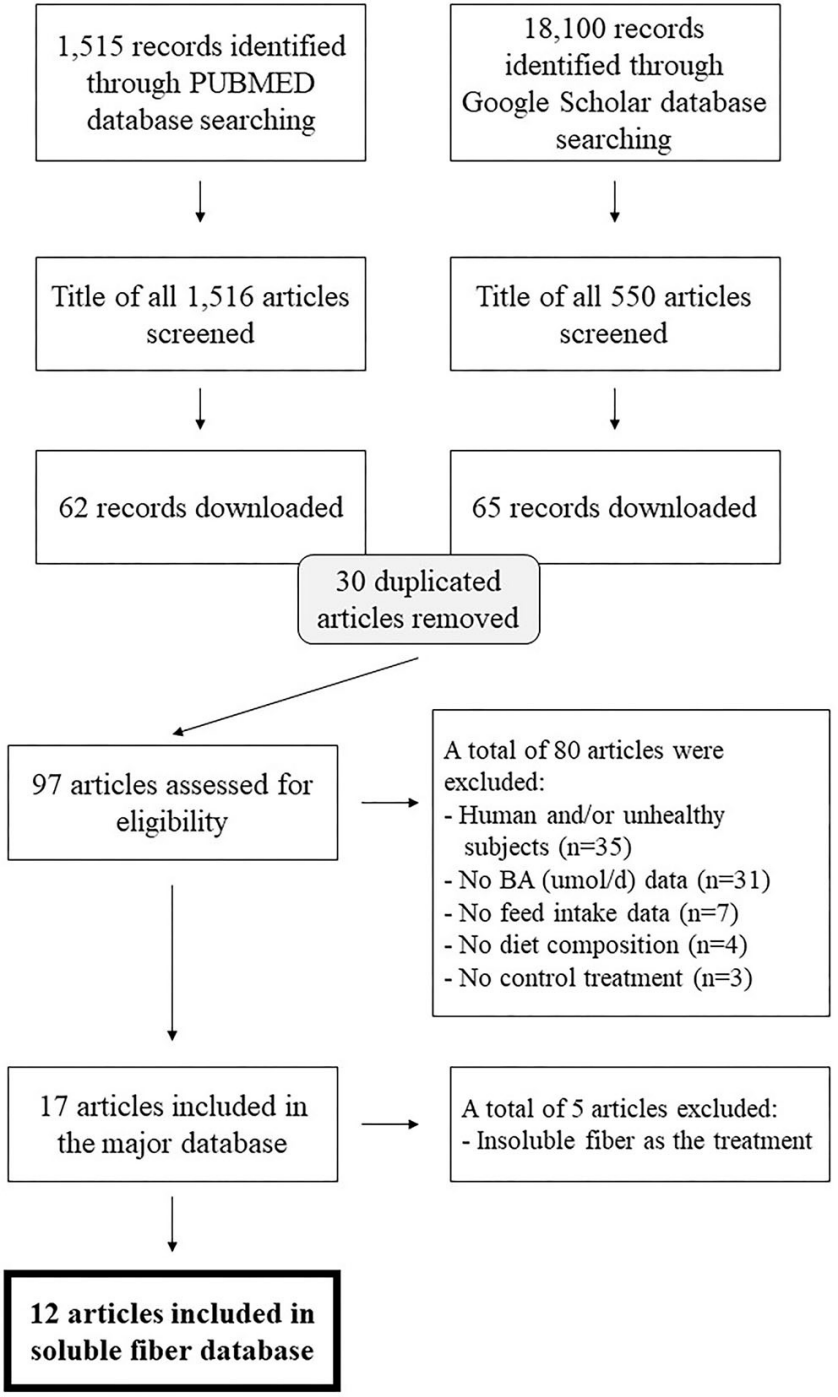
Literature funnel.

A systematic literature review of English language papers published after 1985 was conducted in two databases: PubMed and Google Scholar. The search was conducted between February and March 2021. The following terms were used to identify studies that measure the effect of fiber on total excretion of fecal bile acids: “bile acid [title] OR bile acids [title] AND fiber OR fiber AND excretion [title/abstract]” and “fiber AND bile acid AND excretion AND fecal.” This search retrieved 1,515 records in PUBMED and 18,100 records in Google Scholar, as illustrated via literature funnel (Figure 1). After removal of duplicated articles, 97 articles were assessed for eligibility. For inclusion in the dataset, studies were required to include a control treatment group that did not include any major soluble fiber ingredient in the diet, and a soluble fiber treatment that consisted of a diet with inclusion of a soluble fiber source (e.g., pectin). In addition, articles must have used monogastric species as subjects and reported the following: feed intake, fecal excretion of BA (μmol/d) and the ingredient or nutritional composition of the diet. Studies that did not report BA in μmol/d were considered eligible if sufficient data was reported to allow conversion of BA from the reported unit to μmol/d. If individual BA output was reported, they were summed up and added in the database as total excretion of BA. In addition, studies on humans and/or unhealthy subjects were excluded (the former, due to the lack of data on feed intake and diet composition, the latter for potential interaction with the main effects of interest). When ingredient composition of the diet was disclosed, the dietary carbohydrate, fat and protein content (%, as-fed) were calculated according to the ingredient inclusion and its nutritional profile based on the United States Department of Agriculture FoodData Central. Of the 97 articles, ten studies met the aforementioned exclusion criteria and were used for this meta-analysis after outlier removal which led to a total of 30 treatment means from 278 animals from four species [(rat = 5), hamster (*n* = 1), guinea pig (*n* = 3) and dog (*n* = 1)]. Of the 30 treatment means, 7 represented control treatments and 23 soluble fiber treatments (due to multiple soluble fiber treatments/study) (Table 1).

**Table 1:** Summary description of publications included in the meta-analysis.

**References**	**Species**	**Experimental^**a**^Design**	**N^**b**^**	**Weeks on treatment**	**Cholesterol inclusion**	**Soluble fiber source (SFC)^**c**^**	**SFC Inclusion (%)**	**Results^**d**^**
Gallaher and Chen (21)	Rat	CRD	30	6	N	Soy polysaccharide	10.36	=^e^
Innami et al. (22)	Rat	RCBD	32	3.5	N	Jews Mellow Leaf	15.02	↑
						Persimmon Leaf	10.62	↑
Kim and Shin (23)	Rat	CRD	32	4	Y	Chicory Extract	1	↑
						Chicory Extract	5	↑
						Inulin	5	↑
Ko et al. (19)	Dog	RCBD	12	12	N	Beet Pulp	4.7	↑
Levrat et al. (24)	Rat	CRD	20	3	N	Inulin	10	↑
Moundras et al. (25)	Rat	CRD	36	3	N	Gum Arabic	7.5	↑
						Guar Gum	7.5	↑
Santas et al. (26)	Guinea Pig	CRD	16	8	N	Maltodextrin	12	=
Terpstra et al. (27)	Hamster	RCBD	56	7	Y	Calcium-sensitive pectin	3	=
						Non-calcium sentitive-pectin	3	=
						Psyllium	3	↑
Trautwein et al. (28)	Hamster	CRD	30	4	Y	Low-esterificaiton pectin	8	=
						High-esterificaiton pectin	8	=
						High viscosity guar gum	8	=
						Low viscosity guar gum	8	=
Trautwein et al. (29)	Hamster	CRD	40	6	Y	High-esterificaiton Pectin	8	=
						Low-esterificaiton Pectin	8	=
						High viscosity guar gum	8	=
						Low viscosity guar gum	8	=

a *CRD, complete randomized design; RCBD, randomized complete block design*.

b *Total number of animals in the control and treatment group*.

c *Only the treatments included in the model after outlier removal are presented*.

d *Results from total excretion of bile acids from the treatment group compared to control, where: = no effect; ↑ higher compared to the control group; ↓ lower compared to the control group*.

e *Compared to the high-fat control group*.

For data extraction and database development, additional information such as the addition of cellulose or cholesterol to the diet, as well as the duration of each dietary treatment, was recorded. In experiments where total excretion of BA was reported at multiple time-points, only the means for the last time point were added to the database. One study provided total excretion of BA data via graph, and Web Plot Digitizer was used to extract those values (WebPlotDigitizer, version 4.4). Because eligible studies contained different species as subjects, total excretion of BA was standardized to the feed intake to allow comparison between species:


(1) $$\begin{array}{l} \text{Total fecal BA excretion}(\text{μ mol/g feed intake}):\frac{\text{total fecal BA excretion}(\frac{\text{μ mol}}{\text{d}})}{\text{feed intake }(\frac{\text{g}}{\text{d}})} \end{array}$$

Descriptive analysis of the dependent and the independent variables in the dataset are reported in Table 2.

**Table 2:** Descriptive statistics of the dependent and independent variables in the dataset.

**Variable^**a**^**	**All data**			**Rats**	**Hamster**	**Dog**	**Guinea Pig**
	**Mean (SD)**	**Min**	**Max**	**Mean (SD)**	**Mean (SD)**	**Mean (SD)**	**Mean (SD)**
**Total fecal excretion of BA (μmol/d)**							
Control	19.9 (36.57)	2.7	103	7.94 (2.912)	3.25 (0.771)	102	6.29
Soluble Fiber	16.4 (38.17)	2.7	189	13.9 (5.47)	3.69 (0.856)	189	7.61
Overall	17.2 (37.21)	2.7	189	12.5 (5.54)	3.62 (0.829)	146 (61)	6.95 (0.933)
**Total fecal excretion of BA (μmol/g feed intake)**							
Control	0.30 (0.121)	0.18	0.50	0.40 (0.130)	0.22 (0.055)	0.24	0.23
Soluble Fiber	0.44 (0.273)	0.17	1.07	0.68 (0.240)	0.23 (0.071)	0.43	0.29
Overall	0.41 (0.251)	0.17	1.07	0.62 (0.248)	0.23 (0.067)	0.33 (0.132)	0.26 (0.043)
Feed intake (g/day)	46.4 (105.58)	13.8	443	20.3 (2.66)	15.9 (1.67)	435 (12)	26.9 (0.85)
Dietary carbohydrate (%)	54.8 (8.71)	8.71	72	61.6 (8.29)	49.5 (4.01)	55.8 (1.40)	44.3
Dietary fat (%)	16.2 (5.59)	6.31	21.1	15.5 (5.09)	16.1 (6.80)	11.8 (0.07)	16.9
Dietary protein (%)	14.1 (4.04)	6.2	18.1	13.1 (4.89)	14.8 (3.21)	20.3	18.1
*n* (treatment means)	30	–	–	13	13	2	2
*n* (studies)	10	–	–	5	3	1	1

a *BA = bile acids; Overall = control and soluble fiber treatment results combined*.

### Model Development

All procedures were performed using SAS studio (SAS Inst., Cary, NC). The following driving variables (X) were assessed to model total fecal BA excretion (μmol/g intake): dietary carbohydrate (% of feed, as-fed), dietary fat (% of feed, as-fed), dietary protein (% of feed, as-fed), cellulose inclusion (yes/no and continuous), cholesterol inclusion (yes/no and continuous), soluble fiber inclusion (yes/no and continuous), time on treatment (weeks) and species. Cellulose, cholesterol and soluble fiber were evaluated as both categorical (yes/no, included or not included) and continuous (inclusion level, % of feed, as-fed) variables. The PROC CORR procedure was used to evaluate collinearity between continuous driving (X) variables, and was claimed when *P* < 0.05 for the correlation. Variables that were collinear were not considered for placement in the same equations together.

To visualize the database, the mean difference (MD) was calculated by subtracting the total BA excretion (μmol/g feed intake) in the control group from the soluble fiber group in each study. The SEM of the MD was used to calculate the 95% confidence internal of the MD and to generate a forest plot using PROC SGPLOT.

After visual assessment of the data and model assumptions, data were log-transformed for analysis and model development. Mixed models were developed using the PROC GLIMMIX procedure of SAS. Study was treated as a random effect to account for between-study differences with respect to the predicted and driving variables between studies (30). First, univariate models considering soluble fiber (categorical variable) were developed. Next, the driving variables (Xs) related to diet composition were assessed within univariate models or as an expansion of the soluble fiber univariate model. Finally, the effect of species was assessed in the models that considered soluble fiber and the driving variables related to diet composition to develop multivariate models that describe variation in the data due to both diet composition and species. Variables were retained as fixed effects in the model when deemed significant (*P* < 0.05) and where the residual error and random effect were normally distributed. Model assumptions regarding the residuals and random effect were evaluated via visual assessment of residual, histogram and QQ plots and using the Shapiro-Wilk test. The corrected Akaike information criterion (AICc) was used for model comparison to evaluate model goodness of fit during development (to compare between models and within models to determine the optimal variance/covariance structure and inclusion/exclusion of random/fixed effects), where lower values indicate better fit. Influential outliers were detected and removed from the dataset using the Cook's distance test in PROC MIXED. A summary of the dataset after outlier removal is provided in Table 1.

### Model Evaluation

Models were evaluated both visually and statistically. Plots of predicted vs. observed and predicted vs. conditional residuals (predicted – observed) were generated for visual assessment of patterns after model development. When a driving variable was deemed non-significant and removed, the removed variable was plotted vs. the residuals to further investigate if it influenced the model. To do so, significance of the slope (*P* < 0.05) of the residual vs. variable plot was evaluated through the PROC REG procedure of SAS.

The models developed were statistically evaluated back on the model development database by calculating the mean square prediction error (MSPE) as follow:


(2) $$\begin{array}{l} MSPE=\sum_{i=1}^{n}{\left(Oi-Pi\right)}^{2}/n \end{array}$$

Where *Oi* is the observed value, *Pi* is the predicted value and n is the number of observations. The MSPE was also expressed as the square root of MSPE (RMSPE), relative to the observed mean (%), as:


(3) $$\begin{array}{l} RMSPE=\frac{\sqrt{MSPE}}{\bar{O}}*100 \end{array}$$

The RMSPE was decomposed into error due to random disturbance (ED), overall bias error (ECT) and regression slope deviation (ER) according to Bibby and Toutenburg (31). Each category was expressed as percentage of MSPE and calculated as follow:


(4) $$\begin{array}{l} ECT={\left(\bar{P}-\bar{O}\right)}^{2} \end{array}$$

(5) $$\begin{array}{l} ED=\left(1-R^{2}\right)*So^{2} \end{array}$$

(6) $$\begin{array}{l} ER={\left(Sp-R*So\right)}^{2} \end{array}$$

Where *Sp* is the predicted standard deviation, *So* the observed standard deviation and R the Pearson correlation coefficient.

The concordance correlation coefficient (CCC) was also used for model evaluation, as an index of reproducibility, with value ranging from −1 to 1, where 1 indicates perfect agreement between observed and predicted values, −1 represents perfect disagreement and 0 indicates no agreement (32):


(7) $$\begin{array}{l} CCC=R*Cb \end{array}$$

Where *Cb* (value range 0–1) was calculated as a bias correction factor used to measure accuracy:


(8) $$\begin{array}{l} Cb=\left[\frac{2}{v+\frac{1}{v}+\;u2}\right],wherein: \end{array}$$

(9) $$\begin{array}{l} v=\frac{so}{sp} \end{array}$$

(10) $$\begin{array}{l} u=\frac{\bar{O}-\bar{P}\;}{\sqrt{\left(So*Sp\right)}} \end{array}$$

Equation (9) (*v*) is used to indicate scale shift while equation 10 (*u*) provides an indication of location shift. Wherein, the ideal *v*-value = 1 (values larger than 1 signal a greater variance in the predicted values compared to the observed ones) and a positive or negative *u* value indicates either under or over prediction, respectively.

## Results

**Figure 2:**
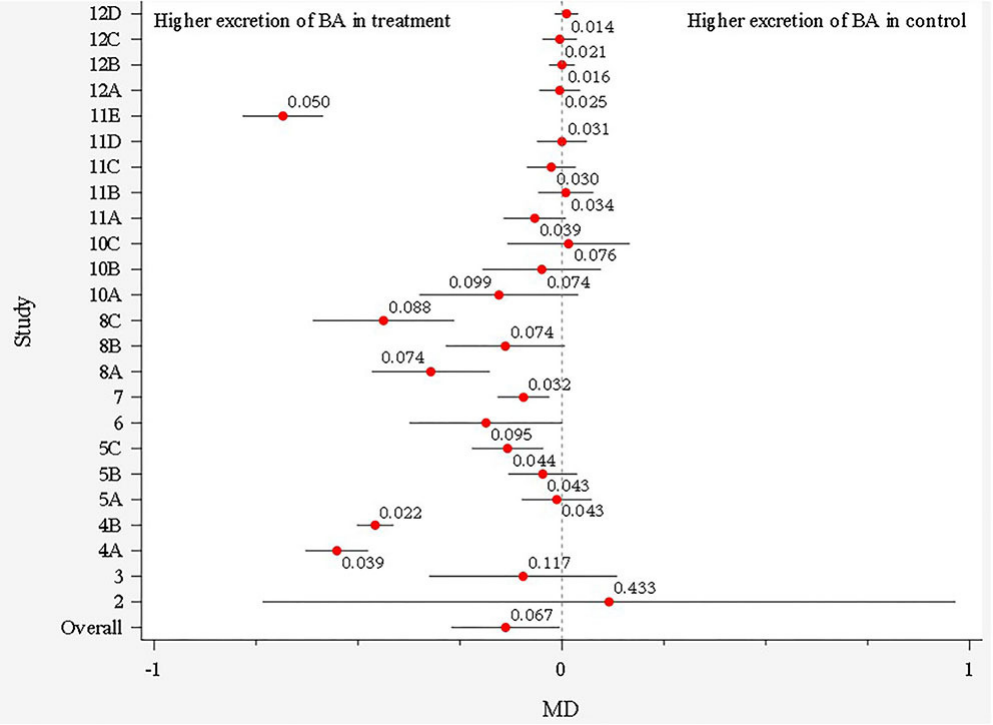
Forest plot showing the main difference (MD) and its 95% CI for each study before outlier removal. Each study is represented by a number and letters represent different soluble fiber treatments within each study.

The soluble fiber treatment MD estimates from studies before outlier removal are illustrated in Figure 2 using a forest plot. One study was removed from this analysis prior to plotting due to extreme values, which were later detected as outliers, and another other study was not included in the plot due to lack of SEM reporting (therefore could not generate 95% confidence interval). The overall MD indicates a possible effect of the soluble fiber treatment on total excretion of BA, favoring greater excretion of the BA in the soluble fiber treatment compared to the control (0.067, lognormal scale, Figure 2). For further model development, individual treatment means were considered rather than MD; wherein, a total of nine observations (treatment means) were detected as influential outliers though the Cook's distance test, and, thus, were removed from the database.

The initial results on correlation between continuous driving variables are presented in Table 3. Dietary carbohydrate was significantly related to dietary protein (*R* = −0.38521; *P* < 0.05) and tended to be related dietary fat (*R* = −0.32295; 0.1 > *P* > 0.05), indicating collinearity between variables. Thus, these driving variables were not combined in model development, and three independent models using either dietary carbohydrate, protein or fat were developed and presented in detail below. Parameter estimates for the model equations can be found in Table 4.

**Table 3:** Correlation analysis between dietary carbohydrate, protein, and fat.

**Variables**	**Dietary carbohydrate**	**Dietary protein**	**Dietary fat**
Dietary Carbohydrate	1.0	–	–
Dietary Protein	−0.385^*^	1.0	
Dietary Fat	−0.323	−0.257	1.0

* *P < 0.05*.

**Table 4:** Parameters estimates for the model equations developed to estimate total bile acid excretion (lognormal data scale).

**Variable**	**Carbohydrate model**		**Protein model**		**Fat model**	
	**Estimate**	***P*** **-value**	**Estimate**	***P*** **-value**	**Estimate**	***P*** **-value**
Intercept	0.934 ± 0.7463	0.2575	−0.954 ± 0.2888	0.0164	−0.897 ± 0.2692	0.0157
Soluble Fiber		0.0027		0.0063		0.0109
No	−0.027 ± 0.0120		0.014 ± 0.0177		0.013 ± 0.0162	
Yes	−0.023 ± 0.0121		0.032 ± 0.0190		0.026 ± 0.0162	
Species		0.0003		0.0006		0.0009
Dog	−0.654 ± 0.2753		−0.454 ± 0.2997		−0.631 ± 0.3148	
Guinea Pig	−1.167 ± 0.3367		−0.821 ± 0.3030		−0.786 ± 0.3043	
Hamster	−1.222 ± 0.2180		−0.974 ± 0.1923		−0.955 ± 0.1964	
Rat	0		0		0	

### Carbohydrate Model

The interaction between dietary carbohydrate content (continuous) and soluble fiber inclusion (categorical) (*P* < 0.05) and the single effect of species (categorical; *P* < 0.05) significantly affected the dependent variable (excretion of BA); thus, they were included as fixed effects in the final carbohydrate model (Table 4). Increasing dietary carbohydrate resulted in lower fecal excretion of BA. The addition of an ingredient that is a major source of soluble fiber resulted in greater fecal excretion of BA. All the other independent/driving variables were not significant (*P* > 0.05), and consequently, were excluded from the model. Analysis of each non-significant independent X variable vs. residuals revealed a constant distribution of residuals and a non-significant slope estimate (*P* > 0.05), indicating no perceivable impact of the excluded independent variable on total excretion BA (data not shown). This was further confirmed through the lack of significance of the slope (*P* > 0.05) of residual vs. excluded variable. The LSmeans for the effect of species were separated using Fischer's LSD; wherein, total excretion of BA was greater for rats compared to the other species (*P* < 0.05; Table 5). The eight predicted equations produced for the fat model are shown in Table 6.

**Table 5:** Fecal bile acid (BA) concentrations (lognormal data scale) in dogs, guinea pigs, hamster and rats for the model equations developed.

**Species**	**Carbohydrate model**		**Protein model**		**Fat model**	
	LSmeans^*^	***P*** **-value**	LSmeans^*^	***P*** **-value**	LSmeans^*^	***P*** **-value**
Dog	−1.11^b^ ± 0.244	0.0003	−1.08^ab^ ± 0.300	0.0006	−1.217^ab^ ± 0.284	0.0009
Guinea Pig	−1.62^b^ ± 0.275		−1.45^b^ ± 0.303		−1.372^b^ ± 0.304	
Hamster	−1.68^b^ ± 0.146		−1.60^b^ ± 0.192		−1.540^b^ ± 0.196	
Rat	−0.46^a^ ± 0.134		−0.63^a^ ± 0.121		−0.586^a^ ± 0.125	

* *Fecal excretion of BA must be backtransformed from a lognormal distribution to obtain results in μmol/g food intake*.

**Table 6:** Carbohydrate model equations for total fecal bile acids excretion.

**Species**	**Soluble fiber^**a**^**	**Outcome^**b**^**	**Equation**
Dog	No	Fecal excretion of BA (lognormal distribution)	= 0.934 (± 0.7463) – 0.654 (± 0.275) – 0.0273 (± 0.012) × Dietary carbohydrate (%)
	Yes		= 0.934 (± 0.7463) – 0.654 (± 0.275) – 0.0234 (± 0.012) × Dietary carbohydrate (%)
Guinea Pig	No		= 0.934 (± 0.7463) – 1.167 (± 0.337) – 0.0273 (± 0.012) × Dietary carbohydrate (%)
	Yes		= 0.934 (± 0.7463) – 1.167 (± 0.337) – 0.0234 (± 0.012) × Dietary carbohydrate (%)
Hamster	No		= 0.934 (± 0.7463) – 1.222 (± 0.218) – 0.0273 (± 0.012) × Dietary carbohydrate (%)
	Yes		= 0.934 (± 0.7463) – 1.222 (± 0.218) – 0.0234 (± 0.012) × Dietary carbohydrate (%)
Rat	No		= 0.934 (± 0.7463) – 0.0273 (± 0.012) × Dietary carbohydrate (%)
	Yes		= 0.934 (± 0.7463) – 0.0234 (± 0.012) × Dietary carbohydrate (%)

a *Addition of an ingredient in the diet that is a major source of soluble fiber*.

b *Fecal excretion of BA must be backtransformed from a lognormal distribution to obtain results in μmol/g food intake*.

### Protein Model

The interaction between dietary protein content (continuous) and soluble fiber inclusion (categorical) (*P* < 0.05) and the single effect of species (categorical) (*P* < 0.05) significantly affected the dependent variable (excretion of BA); thus, they were included as fixed effects in the final protein model (Table 3). Increasing dietary protein resulted in greater fecal excretion of BA that was enhanced by the addition of a soluble fiber ingredient. Similar to the carbohydrate model, all the other independent variables were not significant, and were excluded from the final model. Analysis of each non-significant independent X variable vs. residuals reveled a constant distribution of residuals and a non-significant slope estimate (*P* > 0.05), indicating no perceivable impact of the excluded independent variable on total excretion BA (data not shown). This was further confirmed through the lack of significance of the slope (*P* > 0.05) of residual vs. excluded variable. The LSmeans for the effect of species were separated using Fischer's LSD. Total excretion of BA was greater for rat compared to hamster and guinea pig (*P* < 0.05), while dogs presented similar excretion of BA compared to the other species (*P* > 0.05). The eight predicted equations produced for the protein model are shown in Table 7.

**Table 7:** Protein model equations for total fecal bile acids excretion.

**Species**	**Soluble fiber^**a**^**	**Outcome^**b**^**	**Equation**
Dog	No	Fecal excretion of BA (lognormal distribution)	= -0.954 (± 0.2888) – 0.454 (± 0.2997) + 0.014 (± 0.0177) × Dietary protein (%)
	Yes		= -0.954 (± 0.2888) – 0.454 (± 0.2997) + 0.032 (± 0.0190) × Dietary protein (%)
Guinea Pig	No		= -0.954 (± 0.2888) – 0.821 (± 0.3030) + 0.014 (± 0.0177) × Dietary protein (%)
	Yes		= -0.954 (± 0.2888) – 0.821 (± 0.3030) + 0.032 (± 0.0190) × Dietary protein (%)
Hamster	No		= -0.954 (± 0.2888) – 0.974 (± 0.1923) + 0.014 (± 0.0177) × Dietary protein (%)
	Yes		= -0.954 (± 0.2888) – 0.974 (± 0.1923) + 0.032 (± 0.0190) × Dietary protein (%)
Rat	No		= -0.954 (± 0.2888) + 0.014 (± 0.0177) × Dietary protein (%)
	Yes		= -0.954 (± 0.2888) + 0.032 (± 0.0190) × Dietary protein (%)

a *Addition of an ingredient in the diet that is a major source of soluble fiber*.

b *Fecal excretion of BA must be backtransformed from a lognormal distribution to obtain results in μmol/g food intake*.

### Fat Model

All independent variables were tested for their significance in predicting total fecal excretion of BA. The interaction between dietary protein content (continuous) and soluble fiber inclusion (categorical) (*P* < 0.05) and the single effect of species (*P* < 0.05) significantly affected the dependent variable; thus, they were included as fixed effects. Increasing dietary fat resulted in greater fecal excretion of BA that was enhanced by the addition of a soluble fiber ingredient. Similar to the carbohydrate and protein model, all the other independent variables were not significant, and were removed from the model. Analysis of each non-significant independent variable vs. residuals revealed a constant distribution of residuals, indicating no impact of the independent variable on total excretion of BA (data not shown). This was further confirmed through the lack of significance of the slope (*P* > 0.05) of residual vs. excluded variable. The LSmeans for the effect of species were separated using Fischer's LSD. Similar to the protein model, total excretion of BA was greater for rats compared to hamsters and guinea pig (*P* < 0.05), while dogs presented similar excretion of BA compared to the other species (*P* > 0.05). The eight predicted equations produced for the fat model are shown in Table 8.

**Table 8:** Fat model equations for total fecal bile acids excretion.

**Species**	**Soluble fiber^**a**^**	**Outcome^**b**^**	**Equation**
Dog	No	Fecal excretion of BA (lognormal distribution)	= -0.897 (±0.2692) – 0.631 (±0.3148) + 0.013 (±0.0162) × Dietary fat (%)
	Yes		= -0.897 (±0.2692) – 0.631 (±0.3148) + 0.026 (±0.0162) × Dietary fat (%)
Guinea Pig	No		= -0.897 (±0.2692) – 0.786 (±0.3043) + 0.013 (±0.0162) × Dietary fat (%)
	Yes		= -0.897 (±0.2692) – 0.786 (±0.3043) + 0.026 (±0.0162) × Dietary fat (%)
Hamster	No		= -0.897 (±0.2692) – 0.955 (±0.1964) + 0.013 (±0.0162) × Dietary fat (%)
	Yes		= -0.897 (±0.2692) – 0.955 (±0.1964) + 0.026 (±0.0162) × Dietary fat (%)
Rat	No		= -0.897 (±0.2692) + 0.013 (±0.0162) × Dietary fat (%)
	Yes		= -0.897 (±0.2692) + 0.026 (±0.0162) × Dietary fat (%)

a *Addition of an ingredient in the diet that is a major source of soluble fiber*.

b *Fecal excretion of BA must be backtransformed from a lognormal distribution to obtain results in μmol/g food intake*.

### Model Evaluation

**Figure 3:**
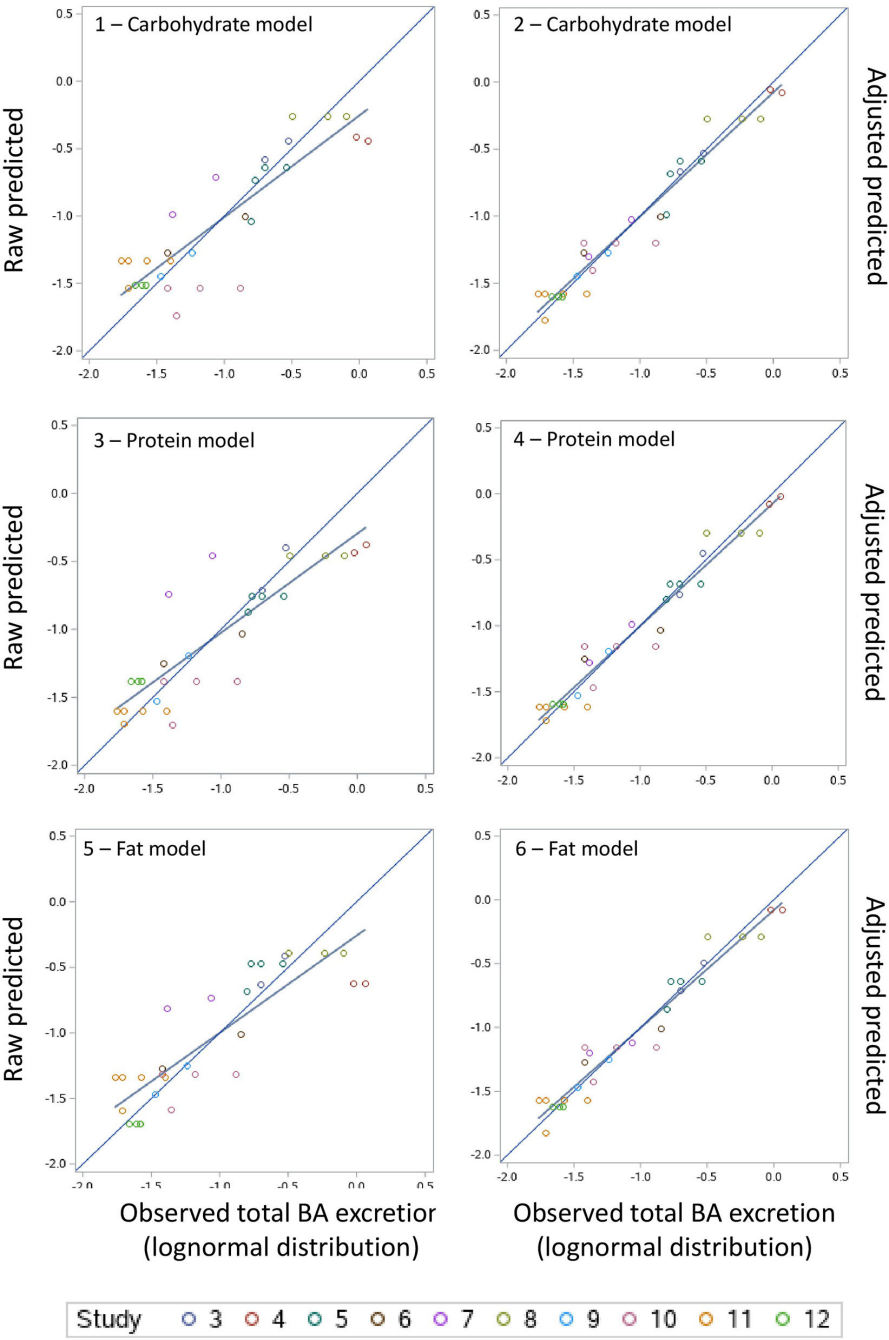
Raw predicted vs. observed and adjusted-predicted vs. observed plots for the carbohydrate, protein and fat models.

**Figure 4:**
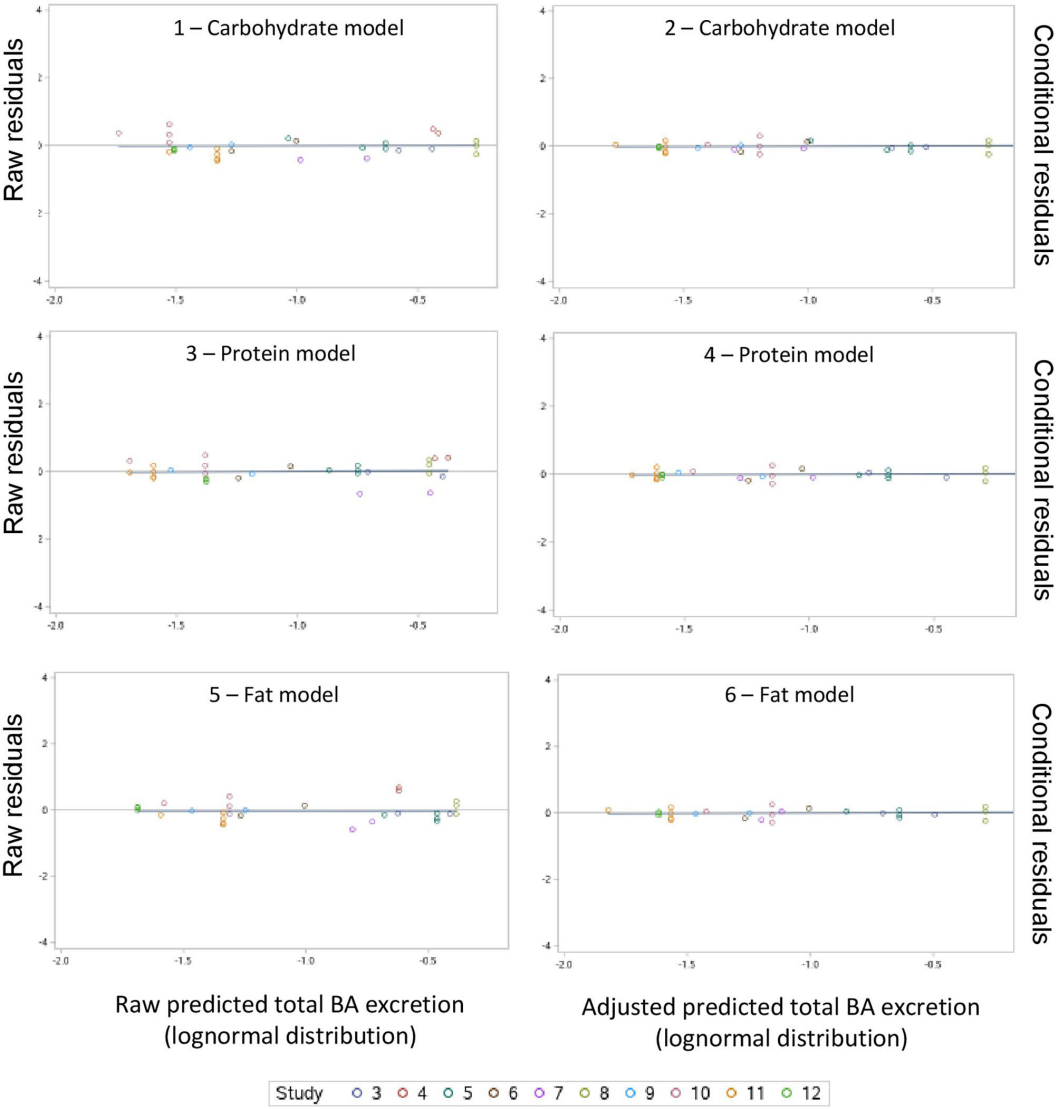
Raw residuals (predicted – observed) vs. raw predicted and conditional residuals vs. adjusted predicted plots for the carbohydrate, protein and fat models.

A summary of the model evaluation statistics for the three models, based on both raw (based on the fixed effect portion of the model) and adjusted (based on the fixed and random portions of the model) Y and residual values, are presented in Table 9. For the three models, adjusted values consistently, and as expected, resulted in a better model prediction compared to the raw values due to the extra consideration of the random effect of study in the calculation of adjusted Y values. The adjusted predictions speak to overall model goodness-of-fit, and the latter to the amount of variation described by the fixed effects model. According to the AICc values, equations based on the carbohydrate model had a better fit followed by the protein and fat models. A similar pattern was observed for the other model evaluation statistics evaluated (RMSPE, ER, ED, ECT, CCC, R, Cb, *v* and u). When adjusted values were used for model evaluation, a near-perfect average prediction of the observed values was revealed (*u* = 0). The RSMPE ranged roughly between 25 and 26% for raw predictions and the breakdown of the RSMPE reveals that more than 99% of it is attributable to random error. The CCC values were close to 1.0 in each model with R and Cb values indicating high precision and accuracy, respectively. The *u*-values indicate a minor over prediction of total excretion of BA in the protein model, while a minor under prediction was observed for the carbohydrate and fat model when raw values were used. The *v*-values indicated a slightly greater variance in the observed values compared to the predicted ones in all models when raw values were used. Visual assessment of the predicted vs. observed (Figure 3) and predicted vs. residuals plots (Figure 4) demonstrate excellent model fit when adjusted values were used compared to slightly more variable raw values, which supports the aforementioned results. However, the slope of the predicted vs. residuals when both raw and predicted values were used was not different from zero in all models (*P* > 0.05). This indicates that predicted and observed values were similar to each other even when raw values were used; thus, the use of raw values in the models still provides a good prediction of the dependent variable.

**Table 9:** Evaluation of model equations using adjusted and raw residuals for total fecal excretion of bile acids.

**Evaluation parameter**	**Fat model**		**Protein model**		**Carbohydrate model**	
	**Adjusted**	**Raw**	**Adjusted**	**Raw**	**Adjusted**	**Raw**
N^a^	30	30	30	30	30	30
AICc^b^	16.63	16.63	15.18	15.18	13.08	13.08
RMSPE (%)^c^	−11.98	−26.26	−11.70	−25.44	−11.79	−24.95
ER (%)^c^^,^^d^	0	0.33	0	0.12	0	0.04
ED (%)^e^	1.05	0	1.00	0.42	0.92	0.07
ECT (%)^f^	98.95	99.67	99.00	99.46	99.08	99.89
CCC^g^	0.9715	0.8505	0.9729	0.8567	0.973	0.8653
R^h^	0.967	0.824	0.9685	0.8302	0.9689	0.8565
Cb^i^	0.999	0.988	0.999	0.984	0.999	0.989
V^j^	1.054	1.1608	1.051	1.1932	1.051	1.1596
U^k^	0	−0.0314	0	0.0187	0	−0.0110

a *Sample size*.

b *Corrected Akaike information criterion as a measure of goodness-of-fit*.

c *Square root of the mean square prediction error expressed as percentage of the observed mean*.

d *Regression slope deviation error expressed as percentage of mean square prediction error (MSPE)*.

e *Rando disturbance error expressed as percentage of MSPE*.

f *Overall bias error expressed as percentage of MSPE*.

g *Concordance correlation coefficient*.

h *Pearson correlation*.

i *Bias correction factor to measure accuracy*.

j *Indicative of scale shift*.

k *Indicative of location shift*.

## Discussion

This study aimed to investigate the effects of soluble fiber, macronutrients and species on total fecal excretion of BA. Such effects have been previously investigated separately in different studies; however, this is the first attempt to use a meta-analysis approach to systematically quantify the interaction of dietary components and species on fecal excretion of BA.

### Effects of Soluble Fiber on Excretion of BA

As hypothesized, inclusion of an ingredient that is a major source of soluble fiber content in the diet resulted in greater excretion of BA. Some fibers can bind BA (33, 34), which prevents reabsorption in the distal ileum, and increases loss via feces. Moreover, the end-product of microbial fermentation of soluble fibers in the gut impact luminal pH (35), which can affect the solubility and excretion of BA. Indeed, the gut microbiome plays a major role in the metabolism of BA, and deserves further consideration. Primary bile acids are produced in the hepatocytes from cholesterol and are released in the small intestine to aid with fat digestion. Some microbial species found in the small intestine can produce bile salt hydrolase that cleave the amino bond between taurine or glycine and the primary bile salt (36). These unconjugated BA cannot be reabsorbed through the enterohepatic circulation. Consequently, they end up in the large intestine where they can undergo further bacterial transformation leading to the production of secondary BA. The majority of secondary BA are excreted in the feces (9). Any dietary factors, such as soluble fibers, that have a major impact on the gut microbiome should be considered during evaluation of BA metabolism. Unfortunately, there is a dearth of published data investigating both BA metabolism and the gut microbiome. Usually, the ratio of primary to secondary BA in the feces is used as a tool to assess the relationship between the gut microbiome and metabolism of BA. Only one study used in this meta-analysis reported fecal output of primary and secondary BA, which limited our ability to analyze this outcome.

### Effects of Macronutrients on Fecal Excretion of BA

Similar to the inclusion level of the fiber source, the inclusion of cellulose and cholesterol in the diet were not significant, likely due to the small range of inclusions in the studies used in this meta-analysis. However, dietary carbohydrate, protein and fat (%) did play a major role in the total excretion of BA. Increasing levels of dietary protein and fat resulted in greater excretion of fecal BA, which was enhanced by the addition of soluble fiber. Dietary fat and protein are well-known for their effect on the release of BA into the small intestine. Most animals secrete CCK in response to dietary fat and protein; however, mechanisms of nutrient-activated release of CCK are species-specific. For example, while CCK release is stimulated by phenylalanine and tryptophan in dogs, rats required intact proteins to trigger such response (37). Increasing dietary fat intake results in greater excretion of BA in rats (38), humans (39), and monkeys (40). However, the type of fat may further modulate these effects. For example, polyunsaturated fatty acids lead to greater excretion of BA compared to saturated fatty acids. In rats, medium (41), but not long- chain triglycerides (42, 43), stimulate CCK release. Unlike dietary protein, the relationship between dietary fat and fecal excretion of BA is not linear in all species (44). This may help explain the better model fit observed for the protein model compared to the fat model. The carbohydrate model resulted in a better fit compared to the fat and protein models. Similar to the protein and fat models, the addition of soluble fiber in the diet resulted in greater excretion of BA in the carbohydrate model. However, increasing dietary carbohydrate led to lower excretion of fecal BA. This was expected due to the negative correlation between inclusion of carbohydrates in the diet and dietary protein and fat. Except in pigs (45), carbohydrates are weak stimulants of CCK release (42). Furthermore, Bianchini et al. (46) reported that dietary starch did not impact fecal BA excretion in rats; however, it had a binding effect on BA *in vitro*. There are still a lack of studies investigating the effects of starch on fecal excretion of BA. It's noteworthy that in this meta-analysis, dietary carbohydrate was calculated based on the ingredient composition of each study and represents a broad category of compounds (e.g., starch, sugar, dietary fiber, etc.). However, as most diets used in the studies were semi-synthetic, the majority of the calculated carbohydrate content is likely pure starch. Although the carbohydrate model resulted in a better fit, the fat and protein model might be more biologically relevant due to their direct effect on secretion of BA.

### Effects of Species on Fecal Excretion of BA

This study found a significant effect of species on BA excretion in all models. In the fat and protein model, greater fecal excretion of BA was observed in rats compared to guinea pigs and hamsters, but was similar to dogs; while in the carbohydrate model, rats displayed a greater excretion of BA compared to all species. Similar results were observed by Kasbo et al. (47) where they observed not only a difference in BA composition, but also greater BA concentrations in fecal pellets from rats compared to hamsters and guinea pigs. This is likely because rats do not possess a gallbladder, which results in a continuous secretion of diluted bile fluid in large volumes (48). This major species difference may limit the ability to translate the results from rats to other species such as the dog. However, owing to the limited sample size of studies using dogs (*n* = 1) and hamsters (*n* = 1) as subjects in this meta-analysis, the effect of species on total excretion of BA should be considered with caution. Furthermore, differences regarding BA metabolism in a single species of interest may occur. For example, the existence of digestive idiosyncrasies between large and small-breed dogs [e.g., differences in intestinal permeability and transit time; (49)] may result in differences in BA metabolism among breeds. More research investigating BA metabolism in different dog breeds and the effects of dietary components on BA homeostasis, and consequently on taurine losses, is warranted. This paucity of information highlights the need for more research in order to draw sensible conclusions about the development of DCM and BA homeostasis in dogs eating grain-free diets. The current meta-analysis emphasizes that soluble fibers may act in synergy with other dietary components leading to greater fecal excretion of BA. Thus, not only should soluble fiber content be investigated, but also other dietary factors, such as fat and protein, when concerned about BA losses, as well as consideration that effects observed within species may not translate to other species.

### Calculation of BA Excretion

It's noteworthy to discuss how fecal excretion of BA are reported in the literature. Fecal BA can be reported as either total fecal excretion (mass or molar unit/d) or total fecal concentrations (mass or molar unit/g of wet or dry feces). Most types of fibers cause an increase in fecal bulk, and may cause a dilution effect on fecal concentrations of BA (16). For example, Nyman et al. (50) observed a lower concentration of fecal BA in rats fed five different vegetables compared to the basal fiber-free diet; however, no differences were observed when fecal BA were reported as mg/d. This effect was attributed to the higher fecal output in rats fed diets with inclusion of vegetables. Metabolically speaking, total excretion of BA can provide a better understanding of BA homeostasis, as higher excretions are indicative of higher production of BA and greater utilization of glycine and/or taurine. Thus, we conducted this meta-analysis using studies that report, or allow the calculation, of total BA output (μmol/d) as this is a better biomarker for losses of taurine. However, total BA output was expressed relative to feed intake to account for differences in food and nutrient intake among species. Despite being unusual, fecal excretion of BA has already been reported in the literature relative to dry-matter intake (51). This approach was effective in standardizing the dependent Y variable. This can be observed by the lower range of total BA values in the studies used in this meta-analysis when expressed as μmol/g feed intake compared to μmol/d (Table 2). Many studies were not included in this meta-analysis because fecal concentrations instead of total fecal output of BA were reported. Future research should consider reporting fecal BA in both units to allow better comparison between studies.

### Limitations

In this meta-analysis, soluble fibers were grouped as one major category and analyzed as a categorial variable. However, the effect of soluble fibers on BA excretion is a function of both physical (e.g., particle size) and chemical characteristics [e.g., viscosity and solubility; (16)] of the fiber source. Due to the lack of reporting of this information in the studies found, these effects could not be accounted for in the models developed. This may explain the need to log-transform the data to achieve homoscedasticity of residuals. Furthermore, the unbalanced number of control and soluble fiber treatments (control = 7 and soluble fiber = 27) may also have played a role in heterogeneity of the residuals prior to the log-transformation, but was this way due to individual studies having a single control for multiple treatment groups. Future studies should consider reporting more information regarding the fiber composition and characteristics. We first attempted to investigate the effect of soluble fiber as a continuous variable (proportion of soluble fiber in the diet) as this also plays a direct role in excretion of BA. However, most studies do not report the proportion of soluble fiber in the ingredient of interest, which limited our ability to use soluble fiber as a continuous variable in the models. The inclusion level of the fiber source was tested in the model, but it was not significant, and thus, was excluded. Furthermore, no patterns were observed when inclusion level was plotted against the residuals. Even though inclusion level of the fiber source may logically play a role in the fecal excretion of BA, the lack of significance observed in our models is probably due to the small range of inclusion level in the studies used in this meta-analysis.

## Conclusions

In summary, inclusion of an ingredient that is a major source of soluble fiber in the diet increased total excretion of BA. Similarly, increasing levels of dietary protein and fat resulted in greater fecal excretion of BA, which was enhanced by the addition of a soluble fiber ingredient. On the other hand, increasing dietary carbohydrate led to a lower fecal excretion of BA. Although a better model fit was observed for the carbohydrate compared to the fat and protein models, the latter might be more biologically relevant. Rats presented greater excretion of BA compared to hamsters and guinea pigs in all models and compared to dogs in the carbohydrate model. Future studies should consider reporting more information regarding diet composition and physical and chemical characteristics of fiber sources to allow the development of more complex models to predict total excretion of BA.

## Data Availability Statement

The raw data supporting the conclusions of this article will be made available by the authors, without undue reservation.

## Author Contributions

JP organized the database and wrote the first draft of the manuscript. JP and JE performed statistical analysis. JE and AS reviewed an edited the manuscript. All authors have read and approved the final manuscript.

## Conflict of Interest

The authors declare that the research was conducted in the absence of any commercial or financial relationships that could be construed as a potential conflict of interest.

## Publisher's Note

All claims expressed in this article are solely those of the authors and do not necessarily represent those of their affiliated organizations, or those of the publisher, the editors and the reviewers. Any product that may be evaluated in this article, or claim that may be made by its manufacturer, is not guaranteed or endorsed by the publisher.

## References

[1] Fascetti AJ Reed JR Rogers QR Backus RC. Taurine deficiency in dogs with dilated cardiomyopathy: 12 cases (1997–2001). J Am Vet Med. (2003) 223:1137–41. 10.2460/javma.2003.223.1137 14584743

[2] Pion PD Kittleson MD Rogers QR Morris JG. Myocardial failure in cats associated with low plasma taurine: a reversible cardiomyopathy. Science. (1987) 237:764–8. 10.1126/science.3616607 3616607

[3] Pion PD Kittleson MD Thomas WP Skiles ML Rogers QR. Clinical findings in cats with dilated cardiomyopathy and relationship of findings to taurine deficiency. J Am Vet Med Assoc. (1992) 201:267–74. 1500323

[4] Food and Drug Administration. FDA Investigating Potential Connection Between Diet and Cases of Canine Heart Disease. (2018). Available online at: https://www.fda.gov/animal-veterinary/cvm-updates/fda-investigating-potential-connection-between-diet-and-cases-canine-heart-disease (accessed February 15, 2021).

[5] Torres CL Backus RC Fascetti AJ Rogers QR. Taurine status in normal dogs fed a commercial diet associated with taurine deficiency and dilated cardiomyopathy. J Anim Physiol Anim Nutr. (2003) 87:359–72. 10.1046/j.1439-0396.2003.00446.x 14507418

[6] Nakayama F. Composition of gallstone and bile: species difference. J Lab Clin Med. (1969) 73:623–30. 5813217

[7] Wildgrube HJ Stockhausen H Petri J Füssel U Lauer H. Naturally occurring conjugated bile acids, measured by high-performance liquid chromatography, in human, dog, and rabbit bile. J Chromatogr. (1986) 353:207–13. 10.1016/S0021-9673(01)87090-4 3700515

[8] Washizu T Ikenaga H Washizu M Ishida T Tomoda I Kaneko JJ. Bile acid composition of dog and cat gall-bladder bile. Jpn J Vet Res. (1990) 52:423–5. 10.1292/jvms1939.52.423 2348606

[9] Long SL Gahan CG Joyce SA. Interactions between gut bacteria and bile in health and disease. Mol Asp Med. (2017) 56:54–65. 10.1016/j.mam.2017.06.002 28602676

[10] Di Ciaula A Garruti G Baccetto RL Molina-Molina E Bonfrate L Wang DQ . Bile acid physiology. Ann Hepatol. (2018) 16:4–14. 10.5604/01.3001.0010.5493 29080336

[11] Enright EF Griffin BT Gahan CG Joyce SA. Microbiome-mediated bile acid modification: role in intestinal drug absorption and metabolism. Pharmacol Res. (2018) 133:170–86. 10.1016/j.phrs.2018.04.009 29660405

[12] van de Peppel IP Verkade HJ Jonker JW. Metabolic consequences of ileal interruption of the enterohepatic circulation of bile acids. Am J Physiol Gastrointest Liver Physiol. (2020) 319:G619–25. 10.1152/ajpgi.00308.2020 32938201

[13] Story JA Furumoto EJ Buhman KK. Dietary fiber and bile acid metabolism—an update. In: Kritchevsky D Bonfield C, editors. Dietary Fiber in Health and Disease. Boston: Springer (1997). p. 259–66. 10.1007/978-1-4615-5967-2_27 9361851

[14] Pezzali JG Acuff HL Henry W Alexander C Swanson KS Aldrich CG. Effects of different carbohydrate sources on taurine status in healthy Beagle dogs. J Anim Sci. (2020) 98:skaa010. 10.1093/jas/skaa010 31943028 PMC7007769

[15] Hickman MA Bruss ML Morris JG Rogers QR. Dietary protein source (soybean vs. casein) and taurine status affect kinetics of the enterohepatic circulation of taurocholic acid in cats. J Nutr. (1992) 122:1019–28. 10.1093/jn/122.4.1019 1552356

[16] Kritchevsky D. Influence of dietary fiber on bile acid metabolism. Lipids. (1978) 13:982–5. 10.1007/BF02533860 108494

[17] Story JA Furumoto EJ. Dietary fiber and bile acid metabolism. In: Furda I Brine CJ, editors. Dietary Fiber. New York, NY: Plenum Press (1990). p. 365–74.

[18] Thakare R Alamoudi JA Gautam N Rodrigues AD Alnouti Y. Species differences in bile acids I. Plasma and urine bile acid composition. J Appl Toxicol. (2018) 38:1323–35. 10.1002/jat.3644 29785833

[19] Ko KS Fascetti AJ. Dietary beet pulp decreases taurine status in dogs fed low protein diet. J Anim Sci Technol. (2016) 58:1–10. 10.1186/s40781-016-0112-6 27489723 PMC4971673

[20] Donadelli RA Pezzali JG Oba PM Swanson KS Coon C Varney J . A commercial grain-free diet does not decrease plasma amino acids and taurine status but increases bile acid excretion when fed to Labrador Retrievers. Transl Anim Sci. (2020) 4:txaa141. 10.1093/tas/txaa141 32832860 PMC7433909

[21] Gallaher DD Chen CL. Beef tallow, but not corn bran or soybean polysaccharide, reduces large intestinal and fecal bile acid concentrations in rats. Nutr Cancer. (1995) 23:63–7. 10.1080/01635589509514362 7739916

[22] Innami S Tabata K Shimizu J Kusunoki K Ishida H Matsuguma M . Dried green leaf powders of Jew's mellow (Corchorus), persimmon (Diosphyros kaki) and sweet potato (Ipomoea batatas poir) lower hepatic cholesterol concentration and increase fecal bile acid excretion in rats fed a cholesterol-free diet. Plant Foods Hum Nutr. (1998) 52:55–66. 10.1023/A:1008031028484 9839835

[23] Kim M Shin HK. The water-soluble extract of chicory influences serum and liver lipid concentrations, cecal short-chain fatty acid concentrations and fecal lipid excretion in rats. J Nutr. (1998) 128:1731–6. 10.1093/jn/128.10.1731 9772143

[24] Levrat MA Favier ML Moundras C Rémésy C Demigné C Morand C. Role of dietary propionic acid and bile acid excretion in the hypocholesterolemic effects of oligosaccharides in rats. J Nutr. (1994) 124:531–8. 10.1093/jn/124.4.531 8145075

[25] Moundras C Behr SR Demigné C Mazur A Rémésy C. Fermentable polysaccharides that enhance fecal bile acid excretion lower plasma cholesterol and apolipoprotein E-rich HDL in rats. J Nutr. (1994) 124:2179–88. 10.1093/jn/124.11.2179 7965202

[26] Santas J Espadaler J Mancebo R Rafecas M. Selective *in vivo* effect of chitosan on fatty acid, neutral sterol and bile acid excretion: a longitudinal study. Food Chem. (2012) 134:940–7. 10.1016/j.foodchem.2012.02.211 23107711

[27] Terpstra AHM Lapre JA De Vries HT Beynen AC. Dietary pectin with high viscosity lowers plasma and liver cholesterol concentration and plasma cholesteryl ester transfer protein activity in hamsters. J Nutr. (1998) 128:1944–9. 10.1093/jn/128.11.1944 9808647

[28] Trautwein EA Rieckhoff D Kunath-Rau A Erbersdobler HF. Psyllium, not pectin or guar gum, alters lipoprotein and biliary bile acid composition and fecal sterol excretion in the hamster. Lipids. (1998) 33:573–82. 10.1007/s11745-998-0242-6 9655372

[29] Trautwein EA Kunath-Rau AM Erbersdobler HF. Effect of different varieties of pectin and guar gum on plasma, hepatic and biliary lipids and cholesterol gallstone formation in hamsters fed on high-cholesterol diets. Br J Nutr. (1998) 79:463–71. 10.1079/BJN19980077 9682666

[30] St-Pierre NR. Invited review: Integrating quantitative findings from multiple studies using mixed model methodology. J Dairy Sci. (2001) 84:741–55. 10.3168/jds.S0022-0302(01)74530-4 11352149

[31] Bibby J Toutenburg H. Prediction and Improved Estimation in Linear Models. Chichester: Wiley (1977).

[32] Lawrence I Lin K. A concordance correlation coefficient to evaluate reproducibility. Biometrics. (1989) 45:255–68. 10.2307/2532051 2720055

[33] Greenwald P Lanza E Eddy GA. Dietary fiber in the reduction of colon cancer risk. J Am Diet Assoc. (1987) 87:1178–88. 10.1016/S0002-8223(21)03296-X 3040841

[34] Garcia-Diez F Garcia-Mediavilla V Bayon JE Gonzalez-Gallego J. Pectin feeding influences fecal bile acid excretion, hepatic bile acid and cholesterol synthesis and serum cholesterol in rats. J Nutr. (1996) 126:1766–71. 8683337 10.1093/jn/126.7.1766

[35] De Vadder F Kovatcheva-Datchary P Goncalves D Vinera J Zitoun C Duchampt A . Microbiota-generated metabolites promote metabolic benefits via gut-brain neural circuits. Cell. (2014) 156:84–96. 10.1016/j.cell.2013.12.016 24412651

[36] Begley M Hill C Gahan CG. Bile salt hydrolase activity in probiotics. Appl Environ Microbiol. (2006) 72:1729–38. 10.1128/AEM.72.3.1729-1738.2006 16517616 PMC1393245

[37] Morris JG Rogers QR Kim SW Backus RC. Dietary taurine requirement of cats is determined by microbial degradation of taurine in the gut. In: Huxtable RJ Michalk D, editors. Taurine in Health and Disease. New York, NY: Plenum Press (1994). 10.1007/978-1-4899-1471-2_7 7887289

[38] Reddy BS Weisburger JH Wynder EL. Effects of dietary fat level and dimethylhydrazine on fecal acid and neutral sterol excretion and colon carcinogenesis in rats. J Natl Cancer Inst. (1974) 52:507–11. 10.1093/jnci/52.2.507 4816006

[39] Cummings JH Wiggins HS Jenkins DJA Houston H Jivraj T Drasar BS . Influence of diets high and low in animal fat on bowel habit, gastrointestinal transit time, fecal microflora, bile acid, and fat excretion. J Clin Invest. (1978) 61:953–63. 10.1172/JCI109020 659584 PMC372613

[40] Redinger RN Hermann AH Small DM. Primate biliary physiology. X Effects of diet and fasting on biliary lipid secretion and relative composition and bile salt metabolism in the rhesus monkey. Gastroenterology. (1973) 64:610–21. 10.1016/S0016-5085(73)80133-7 4633721

[41] Douglas BR Jansen JBMJ De Jong AJL Lamers CBHW. Effect of various triglycerides on plasma cholecystokinin levels in rats. J Nutr. (1990) 120:686–90. 10.1093/jn/120.7.686 2366103

[42] Liddle RA Green GM Conrad CK Williams JA. Proteins but not amino acids, carbohydrates, or fats stimulate cholecystokinin secretion in the rat. Am J Physiol. (1986) 251:G243–8. 10.1152/ajpgi.1986.251.2.G243 3740265

[43] Jenkins AP Ghatei MA Bloom SR Thompson RPH. Effects of bolus doses of fat on small intestinal structure and on release of gastrin, cholecystokinin, peptide tyrosine-tyrosine, and enteroglucagon. Gut. (1992) 33:218–23. 10.1136/gut.33.2.218 1541417 PMC1373933

[44] Sanderson SL Gross KL Ogburn PN Calvert C Jacobs G Lowry SR . Effects of dietary fat and L-carnitine on plasma and whole blood taurine concentrations and cardiac function in healthy dogs fed protein-restricted diets. Am J Vet Res. (2001) 62:1616–23. 10.2460/ajvr.2001.62.1616 11592329

[45] Cuber J Bernard C Levenez F Chayvialle J. Intraduodenal infusions of fat, protein, and carbohydrate stimulate the release of intestinal cholecystokinin (cck) in the conscious pig. Reprod Nutr Dev. (1990) 30:267–75. 10.1051/rnd:19900213 2350402

[46] Bianchini F Caderni G Dolara P Fantetti L Kriebel D. Effect of dietary fat, starch and cellulose on fecal bile acids in mice. J Nutr. (1989) 119:1617–24. 10.1093/jn/119.11.1617 2600667

[47] Kasbo J Saleem M Perwaiz S Mignault D Lamireau T Tuchweber B . Biliary, fecal and plasma deoxycholic acid in rabbit, hamster, guinea pig, and rat: comparative study and implication in colon cancer. Biol Pharm Bull. (2002) 25:1381–4. 10.1248/bpb.25.1381 12392101

[48] Kararli TT. Comparison of the gastrointestinal anatomy, physiology, and biochemistry of humans and commonly used laboratory animals. Biopharm Drug Dispos. (1995) 16:351–80. 10.1002/bdd.2510160502 8527686

[49] Weber MP Biourge VC Nguyen PG. Digestive sensitivity varies according to size of dogs: a review. J Anim Physiol Anim Nutr. (2017) 101:1–9. 10.1111/jpn.12507 27045769

[50] Nyman M Schweizer TF Tyrén S Reimann S Asp NG. Fermentation of vegetable fiber in the intestinal tract of rats and effects on fecal bulking and bile acid excretion. J Nutr. (1990) 120:459–66. 10.1093/jn/120.5.459 2160526

[51] Ndou SP Kiarie E Ames N Nyachoti CM. Flaxseed meal and oat hulls supplementation: impact on dietary fiber digestibility, and flows of fatty acids and bile acids in growing pigs. J Anim Sci. (2019) 97:291–301. 10.1093/jas/sky398 30321359 PMC6313103

